# Risky Business: Factor Analysis of Survey Data – Assessing the Probability of Incorrect Dimensionalisation

**DOI:** 10.1371/journal.pone.0118900

**Published:** 2015-03-19

**Authors:** Cees van der Eijk, Jonathan Rose

**Affiliations:** Methods and Data Institute, School of Politics and International Relations, University of Nottingham, Nottingham, United Kingdom

## Abstract

This paper undertakes a systematic assessment of the extent to which factor analysis the correct number of latent dimensions (factors) when applied to ordered-categorical survey items (so-called Likert items). We simulate 2400 data sets of uni-dimensional Likert items that vary systematically over a range of conditions such as the underlying population distribution, the number of items, the level of random error, and characteristics of items and item-sets. Each of these datasets is factor analysed in a variety of ways that are frequently used in the extant literature, or that are recommended in current methodological texts. These include exploratory factor retention heuristics such as Kaiser’s criterion, Parallel Analysis and a non-graphical scree test, and (for exploratory and confirmatory analyses) evaluations of model fit. These analyses are conducted on the basis of Pearson and polychoric correlations. We find that, irrespective of the particular mode of analysis, factor analysis applied to ordered-categorical survey data very often leads to over-dimensionalisation. The magnitude of this risk depends on the specific way in which factor analysis is conducted, the number of items, the properties of the set of items, and the underlying population distribution. The paper concludes with a discussion of the consequences of over-dimensionalisation, and a brief mention of alternative modes of analysis that are much less prone to such problems.

## Introduction

Analysts of survey data are confronted with a variety of conflicting methodological recommendations about whether (or how) to use factor analysis for assessing latent meaning dimensions in sets of Likert-type items. Such items violate the assumption of interval-level measurement of the observed variables, and thus there is a question of whether, or under what circumstances, this leads to substantively misleading results. The literature is not explicit on this matter, and arguments are customarily illustrated with examples (sometimes based on simulated data), without specifying how ‘representative’ these examples are for actual applied research.

In this article we estimate the risks of over-dimensionalising when factor analysing ordinal survey data. We do so by conducting factor analyses on a large number (2400) of simulated sets of uni-dimensional Likert items, which vary systematically in ways that would be relevant for actual applied research: the latent distribution of respondents, the number of items, the level of random noise, the range of positions of the items on the underlying dimension, and the skew of the items. Each simulated dataset is analysed in variety of factor analytic ways: using both Pearson and polychoric correlations, using the most popular and most often recommended factor retention criteria in exploratory factor analysis, and using customary statistical evaluation criteria for exploratory and confirmatory factor models.

Our substantive findings include:

1. When relying exclusively on retention heuristics in exploratory factor analysis to determine the number of factors, we find that K1 (the so-called Kaiser criterion, also known as the ‘eigenvalues >1’ rule) is very prone to over-dimensionalisation. Parallel analysis (PA) is less susceptible to this, but still involves a high risk in particular, quite common, circumstances (particularly when using Pearson correlations, or analysing more than 8 items). The acceleration factor never leads to over-dimensionalisation, but cannot be fully recommended in view of reports of a tendency to under-dimensionalise.
2. The risk of over-factoring when using K1 or parallel analysis is considerably reduced when using polychoric, rather than Pearson correlations. Yet, even then, over-dimensionalisation is still likely to occur with larger numbers of items and a skewed underlying population distribution.
3. Statistical evaluation leads in the overwhelming majority of instances to the rejection of a 1-factor model for truly one-dimensional data, and would thus generally lead to over-dimensionalisation. This holds for both exploratory and confirmatory factor analysis, and irrespective of whether Pearson or polychoric correlations are used.
4. The conditions that lead retention criteria (K1 and PA) to over-factoring are: (a) the nature of the underlying population distribution, with particularly high risks in the case of normal and skewed normal distributions; (b) the number of items in the factor analysis: larger numbers of items yield higher risks, *ceteris paribus*; (c) the spread of the item means or medians: larger spread leads to higher risks of over-factoring; (d) the disparity between items in terms of their skew: the larger these differences, the higher the risk of over-factoring; (e) although relatively weak, we find consistently that the larger the level of random noise in the data the smaller the risk is of over-factoring. The results are broadly similar for statistical model evaluation criteria, although then bimodal and uniform populations are associated with the worst fit (and the highest risk over over-dimensionalisation), and the consequences of the number of items being more equivocal.

## Background

Factor analysis is widely used in the analysis of survey data for exploring latent variables underlying responses to survey items, and for testing of hypotheses about such latent variables. Factor analysis is thus intimately linked to the substantive core of empirical social science: the concepts used to describe and understand the empirical world. However, survey data are ubiquitously categorical, a condition that violates basic assumptions of the factor analysis model (both in its exploratory and its confirmatory forms). This may lead to various kinds of incorrect, biased, or misleading results. Of these, we—and many others, e.g. [1,2,3,4]—consider problems relating to the identification of the number of factors to be the most important.

However, while the extant literature agrees that ordinal data violates the assumptions of factor analysis, there is little agreement about the robustness of factor analytic results for these violations. In this article we systematically estimate the risk of over-dimensionalisation for sets of items that reflect a single latent factor. We do so by analysing a large number of simulated one-dimensional datasets that mimic the kind of survey data most often factor analysed in applied social research, ordered categorical items, also known as Likert-items. These are ordered categorical items, with responses defined by bipolar verbal labels that suggest approximately equidistant gradations symmetrically around a neutral middle category. The most common form has five categories: ‘strongly disagree’, ‘disagree’, ‘neither disagree nor agree’, ‘agree’, and ‘strongly agree’. The middle category is sometimes labelled differently, e.g., ‘not certain’. Other variations include (a) reformulation of the ‘agree’ aspect with a more affective label (e.g., ‘prefer’) or with a more behavioural label (e.g., ‘will certainly do’); (b) absence of a neutral middle response category; (c) extension of the number of response categories to six (without a neutral middle) or seven (including such a neutral category). For pragmatic purposes we focus in this article on items with five response categories.

We find that under exceedingly common circumstances, the probability of arriving at over-dimensionalised solutions is unacceptably high.

We start with a brief review of the actual use of factor analysis on social science survey data, and of current methodological recommendations. From this we derive a limited number of commonly used or often recommended procedures which will be evaluated in in our risk assessment. We then elaborate our research design, the simulation of data, and the analyses performed. We then describe the outcomes of these analyses, and conduct a multivariate analysis to identify the major drivers of the risk of over-dimensionalisation. Our analyses demonstrate the merits and limitations of common practice and of methodological recommendations. We proceed with a brief discussion of implications of our findings for the validity of substantive social research that uses factor analysis of survey data. We end with a note on alternatives to factor analysis that are more appropriate to the analysis of ordered-categorical survey data.

### Factor analysis of social science survey data

Factor analysis is a procedure that accounts for the common variance among a set of items by their linear relations to latent dimensions. This model is causal, such that the latent dimensions are assumed to be the cause of responses on the individual items. Applied researchers frequently confuse factor analysis (FA) and principal components analysis (PCA), as observed by [5,6,7,8,9,10]. The two procedures share indeed some characteristics and are often implemented in a single software procedure. PCA and FA are, however, different models with different epistemological foundations. Statements that “… PCA and common factor analysis will lead to similar substantive conclusions” [11] are often made (see also, e.g., [12], and [13]). Yet, this is demonstrably not the case in many circumstances, cf. [9,14,15,16]. In this article we refrain from a comparison of principal components analysis (PCA) and factor analysis (FA) and refer instead to excellent discussion by [5]. Ironically, however, the problem of correct dimensionalisation is also relevant for those instances where PCA is used for purposes that would in our view actually require FA.

Correlations between survey items constitute the data for factor analysis [17]. For product-moment correlations to adequately reflect relationships, observed variables must be measured at interval level (see also [18,19]). The assumed linearity of relationships with latent variables also requires this. Sometimes survey data indeed yield proper metric data, as in the case of magnitude estimation, cf. [20]. However, the use of these possibilities is rare in most academic or commercial surveys. Likert items, which are ordered-categorical, violate this assumption of interval-level measurement. In spite of this violation, the use of factor analysis for probing such survey data is widespread; examples can be found in highly ranked journals in many disciplines, including political science, sociology, psychology, the health sciences and economics.

The very common practice of factor analysing ordinal data is not surprising. Many textbooks condone or encourage such usage by illustrating factor analytic procedures on survey data with little or no discussion of the risks of using ordered-categorical (rather than interval) data. Pallant, for example, illustrates exploratory factor analysis on the basis of 5-category ordinal survey items without any cautionary note, even after having stated earlier that the method requires “a set of correlated *continuous* variables” ([21], p. 185, emphasis added). Likewise, Byrne illustrates the use of confirmatory factor analysis on a set of Likert items, after a rather perfunctory discussion of ordinal data that suggests that the risks of such analyses are negligible if the number of response categories is five or more, and when the items are not too skewed ([22], pp. 71–2). Many other texts or methods-oriented websites explicitly state that Likert items represent a form of ‘quasi-interval’ measurement that can validly be used in factor analysis (cf. [23], pp. 74–75). Indeed, numerous textbooks, academic encyclopaedias and websites offering methodological advice to applied researchers assert that product-moment correlations can validly be calculated on Likert-type items, (e.g., [24], p. 191; [25], p. 26; [26], p.2). And finally, factor analyses of Likert items are so common in the extant peer-reviewed literature that end-users of statistical methods can hardly be faulted for believing that this practice involves no serious risks.

Yet, the practice of factor analysing Likert type items is not uncontested. One of the problems most often mentioned concerns the kind of correlation to be used. The common, though often implicit assumption that Likert-type items are crude categorisations of underlying continuous variables is not a sufficient justification for using Pearson correlations, as the correlation between these underlying continuous variables is attenuated by categorisation [27,28]. The extent of this attenuation is not uniform, however. The smaller the number of categories, the larger the attenuation, *ceteris paribus*. Additionally, attenuation varies as a function of the (observed) distribution of scores: attenuation is minimal when responses are approximately normally distributed with approximately equal means, and is maximal for item pairs that are skewed in opposite directions. Thus, Flora, LaBrish and Chalmers [17] report a (true) population correlation of 0.75 being observed as 0.25 when the continuous variables are categorised into 5-point items; however, for other item pairs the attenuation was much less severe. This implies that observed product-moment correlations may be quite different from their underlying true values, and thus also the factor structure derived from the observed correlations. This is likely to lead to over-dimensionalisation with factors discriminating between left and right skewed items (known as difficulty factors, cf. [29,30,31]); this risk is particularly large when skew varies strongly between items. Moreover, categorisation of true continua leads necessarily to violations of linearity, which add to the inadequacy of the product-moment correlation to represent the relationship between Likert items ([17], p. 13). Polychoric correlations are often recommended as the appropriate correlation measure to use for factor analysis of ordinal items (for an excellent discussion see [32]). These have been shown to approach the true underlying correlation between the items better than product-moment correlations ([17,33,34]). However, they assume underlying normal distributions, which may in some circumstances be rather bold. Moreover, they are also vulnerable to producing inaccurate results in small samples or when items are strongly skewed ([35,36]).

## The Number of Factors in Exploratory Factor Analysis

The most important decision to be made in factor analysis is about the number of factors. A large variety of heuristics and criteria can be found in the literature and in software; some have rarely ever been used in actual applied research, others are common, some have become regarded as out-dated, others are regarded as best practice. We restrict our discussion to those procedures that are demonstrably very popular in actual applications of exploratory factor analysis (EFA)—K1, scree tests and parallel analysis (PA)—and those that are recommended as superior in the contemporary methodological literature—parallel analysis (again), and statistical model evaluation and model comparison. Other procedures will be mentioned only in passing. K1, scree tests and PA are all procedures based on eigenvalues, which substantively reflect the explanatory importance of the (latent) factors for the (observed) variables. Statistical model evaluations do not consider eigenvalues, but instead assess how well an estimated model corresponds with empirical observations.

### K1 (aka Kaiser criterion)

The number of factors is often decided on the basis of the magnitude of the eigenvalues of the correlation matrix. The most well-known form of this criterion is K1, also known as the Kaiser criterion or the Kaiser-Guttman rule, which holds that only factors with eigenvalues ≥ 1 are retained (for a discussion of its origins, see [37]). Jolliffe argued (in the context of principal component analysis) that this rule might be too severe and suggests a cut-off of 0.7 [38], which has actually been used in a number of factor analytic studies (cf. [39]). Raîche, however, observes that 1.40 appears to be “a threshold value for randomness”, although he does not actually propose it as a criterion [40,41].

For many reasons this criterion (and similar ones) is problematic. These criteria suggest that factors marginally above the threshold are substantively relevant, while those marginally below are not at all. Such an all-or-nothing distinction seems unproductive given the noisy character of empirical survey data, sampling variation, effects of (cumulative) rounding in algorithms, and so forth. Some scholars advocate therefore using confidence intervals of eigenvalues, cf. [4,42]. This approach is very rarely used in factor analysis applications. Another problem with these criteria is that the magnitude of eigenvalues is dependent on the number of items. The sum of eigenvalues increases (*ceteris paribus*) with increasing numbers of items, and thus the strength of K1 and similar criteria varies accordingly [43]. Methodological researchers are virtually unanimous in their rejection of the K1 criterion. From analyses on simulated data they invariably find that the K1 and its variations perform poorly, and much worse than several other decision rules ([2,6,41,44,45,46,47]; and many others).

In spite of all these reasons not to use K1 or similar criteria, it is nevertheless the most frequently used criterion in the actual application of factor analysis in social research [6,7,8,9,10,48]. Various factors contribute to this, including the implementation of K1 as default criterion in popular software packages (e.g., SPSS), the clear-cut nature of the criterion that ‘frees’ the researcher from making seemingly subjective judgements, and the self-perpetuating tendency of ‘standard’ practice.

### Scree tests

A second approach to decide on the number of factors is the use of Cattell’s scree test [49], which aims to identify the point of inflection in a graph depicting the magnitude of eigenvalues from largest to smallest. The factors to the left of the inflection are to be retained, the other ones not. In contrast to eigenvalue-threshold criteria this test is not affected by the number of items. In actual research, the scree test is used quite frequently, although decidedly less often than K1. Unfortunately, the scree test is frequently applied incorrectly by including the inflection point in the ‘meaningful’ set of eigenvalues, instead of as the first non-meaningful one (e.g., [11]). The reliance on visual inspection of a graph is often seen as subjective in equivocal real-life applications. However, the literature shows that the scree test performs well at identifying the correct number of factors underlying simulated data sets (cf. [2,50]).

Recently, non-graphical implementations of this test have been developed [51,52], such as the acceleration factor which is based on the second derivative of the eigenvalue distribution. This makes it much easier for applied researchers to evaluate a scree plot. These non-graphical procedures have been shown to perform considerably better than the K1 criterion and approximately on par with parallel analysis (discussed below), although they have been found to be prone to *under*-dimensionalisation [53].

### Parallel analysis (‘PA’)

A procedure recommended as an alternative to the ‘quick and dirty’ K1 rule and the ‘subjective’ scree test is parallel analysis or PA [54,55]. Just as K1 and scree tests, this procedure also focuses on eigenvalues. It compares the eigenvalues from the actual data with those from multiple simulated random data with the same number of variables and cases. Only those factors are to be retained whose eigenvalues exceed the average of the corresponding simulated eigenvalue. Parallel analysis safeguards against capitalisation on chance in small samples, and against large eigenvalues that can be produced by random data. Studies that compared criteria for factor retention conclude that PA consistently outperforms K1 and scree tests [2,37,47,56,57,58,59]. Cho, Li and Bandalos [60] evaluate Glorfeld’s [61] suggestion that the 95^th^ percentile of the simulated eigenvalue distribution should be used, rather than the mean, and find no unequivocal support for using the 95^th^ percentile. However, this more stringent criterion will necessarily mitigate any tendencies of over-dimensionalisation. Usage of PA in applied research has for a considerable period been hampered by paucity of relevant software. However, an increasing number of available software tools for PA have become available in the past decade. These include the module *nFactors* in R [51], which we use in this study, an SPSS macro developed by O’Connor [3] and stand-alone programs [62,63,64]. Moreover, a recently developed interface allows many factor analytic procedures that have been programmed in R to be accessible from within SPSS [65,66]. The popularity of the procedure has no doubt been strongly enhanced by the fact that some high-quality psychology journals recommend the use of PA before considering a manuscript (cf. [67], p. 309). Such semi-compulsory adoption has not occurred elsewhere, and the use of PA in other disciplines still quite limited, particularly when compared to the use of K1 and scree.

### Statistical model evaluation

Most applications of EFA rely exclusively on one (or occasionally on several) of the so-called factor retention criteria discussed above: K1, scree and PA. However, none of these procedures guarantees that an estimated factor model represents the empirical data adequately. If it does not, while a model with a different number of factors does, the latter is to be preferred.

Such statistical evaluation is widely recommended as an (additional) consideration in decisions about the number of factors, but rarely followed in applications of exploratory factor analysis [65].

Statistical evaluation can take various forms. The simplest is by assessing the deviation between the observed correlations and the model’s predictions. Muthén and Muthén recommend that these deviations should (on average) not exceed. 05 [68]. Alternatively, the fit of an estimated EFA model can be assessed with an explicit chi-square test, which evaluates the extent to which the implied correlations of the model are reproduced by the data itself. Here, a non-significant chi-square means that the null hypothesis of a perfectly fitting model cannot be rejected, and therefore that the model is statistically acceptable. Moreover, because a one dimensional EFA model can be conceptually considered to be a proper subset of a two dimensional model, the difference in chi-squares between the one dimensional and two dimensional EFA models can be evaluated directly by a chi-square test of delta chi-square on delta degrees of freedom. If this value is significant there is evidence that the two dimensional model fits significantly better than the one factor model. More generally, the same fit measures can used in EFA that are common in confirmatory factor analysis. Of these the root mean square error of approximation (RMSEA), discussed in more detail below, and root mean square residual are often recommended [63,69], which not only have ‘rule-of-thumb’ cut-offs for model acceptance or rejection but also allow proper testing as their standard errors are known for relevant null-hypotheses (cf. [70,71,72]). More detailed forms of statistical evaluation have also been proposed (e.g., [73]) which focus on local misspecifications that contribute to poor fit, but these go beyond the recommended basic assessment of model fit.

When using statistical evaluation criteria, an initial exploratory factor analytic solution (which may have been obtained by using any of the factor retention criteria mentioned so far) is assessed and, if found to have a poor fit, re-specified until a satisfactory fit has been achieved. One of the most obvious ways of re-specification is to increase the number of factors, which invariably results in better fit.

### Other considerations for factor retention

In addition to those mentioned above, yet other criteria and considerations are sometimes referred to that we do not consider in this study. Velicer’s minimum average partial correlation (MAP) [74] is sometimes mentioned, but it is only appropriate for principal components analysis, not for factor analysis [5]. Another procedure is Revelle and Rocklin’s very simple structure (VSS) [45], although this has not gained much of a foothold in factor analytic applications, and is rarely recommended because little is known about its performance [66].

A non-quantitative consideration that is frequently invoked in introductory texts and methods websites is that resulting factors have to be interpretable and supported by sound theory, or ‘clinical meaningfulness’ ([10], pp.13–14), and that they should be dropped if they are not (cf. [75], p. 822; [5], p. 84). In practice this criterion is indeed referred to frequently by applied researchers, although rarely as a sole criterion. This consideration could conceivably function as a brake against over-dimensionalisation, were it not for the uncanny ability of researchers to ‘interpret’ *any* pattern of factor analytic results. Indeed, Budaev reports an example in which he demonstrates how factors and factor loadings based on random, uncorrelated variables can easily be ‘interpreted’ in terms of the existing literature in his field [9]. We are therefore extremely sceptical of this criterion; particularly in exploratory contexts where it is too easily satisfied by creative researchers to be of any real use.

## The Number of Factors in Confirmatory Factor Analysis

In confirmatory factor analysis (CFA) the number of factors is specified a priori by the researcher on the basis of existing theory and insights. A model specified on that basis is subsequently estimated and subjected to an explicit test of the extent to which the correlations predicted by the model conform to the actual data. Depending on the outcome, the postulated model is tested. If it is rejected, a variety of auxiliary results (such as modification indices, and significance tests of estimated coefficients) can be used to adapt the model and re-test it. Although CFA is not formally an inductive approach, as EFA is, it thus nevertheless allows a sequence of model adaptations that are likely to result in an adequately fitting model. Indeed, CFA (and structural equation modelling more generally) is increasingly seen as amenable to inductive analysis, often referred to as ESEM (exploratory structural equation modelling) [72,76,77]. The question of determining the number of factors presents itself in the CFA context thus in the form of the fit of a postulated model, which often involves a comparison between models of different dimensionality.

A large number of measures exist to evaluate the empirical quality of a CFA model in terms of statistical tests, goodness of fit measures and similar criteria; each of which with its own strengths and weaknesses. Our earlier discussion of statistical evaluation of exploratory factor analysis is equally relevant in this context. In addition to single-model chi-square tests, which were discussed above, measures such as the root mean square error of approximation (RMSEA), goodness-of-fit index (GFI), and adjusted goodness-of-fit index (AGFI) have proved particularly popular in this context, with corresponding ‘rules of thumb’ for their application. RMSEA is a function of the error of approximation per degree of freedom; higher values thus reflect less well fitting models. Because this statistic is based upon error per degree of freedom, it effectively contains a penalty for lack of parsimony. As a rule of thumb models with RMSEA > 0.10 are considered to be poorly fitting, while values below 0.05 are viewed as acceptable. GFI reflects the relative improvement of fit of the specified model over a baseline independence model, and the AGFI adjusts this value for the number of parameters in the model. The literature is in near unanimous agreement in recommending the use of various tests and criteria in conjunction. Clearly, the use of multiple criteria to evaluate a CFA model increases the chance that not all of them support the same conclusion, but that seems preferable over a black-and-white verdict based on any single, but imperfect, criterion.

## Research Strategy

Estimating the risk that factor analysing Likert items results in over-dimensionalisation requires data of which the true dimensionality is known; we therefore simulate data. To acquire a reliable estimate of these risks, a large number of such data sets are required. Also, in order to help applied researchers to gauge these risks in their real-world data, these simulated data sets must vary in characteristics that have been suggested to affect the likelihood of incorrect dimensionalisation. We therefore simulate a large number of datasets that systematically vary on a wide range of characteristics. We vary the number of items (using sets of 5, 8 and 10 items), and levels of random noise. We also vary the differences in item popularity and skew, because of the well-known phenomenon that over-dimensionalisation may be caused by of ‘popularity’ or ‘difficulty’ factors. Rather than fixing the range of item difficulties and skews, we generate variety by sampling items from a relatively large pool of items, where each item has its own unique location on the underlying dimension. This ensures variance, not restricted to a small number of fixed values, in the item popularities and skews even when all other manipulated factors are held constant. Finally, we also vary the underlying population distribution, to assess whether it affects the likelihood of over-dimensionalisation. We generate 2400 simulated data sets, each of which is subjected to a variety of factor analytic procedures. Results from each analysis on each data set are ‘harvested’ and stored in a database of results. This database forms the foundation for estimating the risk of obtaining incorrect factor analysis results in the various conditions specified.

### Specification of data simulation

Data simulation was conducted in R [78]; the full specification is provided in Appendices S1, S2 and S3 in the form of R script files. These files can be used for replication, or, by adapting the scripts, for simulating data according to different specifications.

**Fig 1:**
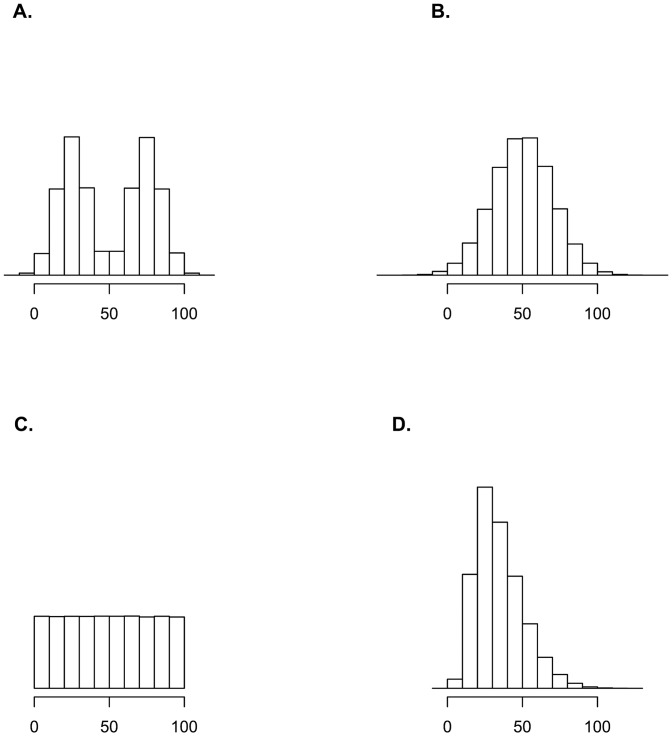
Histograms of simulated respondent positions for four distributions. Histograms of distributions of simulated respondents on the latent continuum used in this paper (n = 2000): bimodal (panel A), normal (panel B), uniform (panel C) and skewed normal (panel D).

We simulate data with a one-dimensional structure. Respondents are characterised by their position on this dimension, which is determined by random draws from a population distribution defined over the dimension. In actual empirical research, the population distribution of a latent variable is, of course, unknown and unknowable (cf. [79]). The only available information is the observed distribution of *responses* to the items. It is obvious (and will be illustrated below) that there is a distinct chasm between these two distributions. Therefore the insistence in many texts on (approximate) normality of response distributions is, in our view, often unproductive. A normally distributed population will not necessarily produce approximately normally distributed responses on categorical items. Moreover, the response distribution may change dramatically when the same items are responded to by a population that is differently distributed, while the functional relationship between the underlying respondent dimension and the responses to the items remains the same. In other simulation studies (e.g., [2,47,54,56,57,60]), and as a popular substantive assumption, it is often postulated that unknown population distributions are normal. Rice for example, stated as far back as 1928 that “there seems to be no obvious *a priori* reason to suppose that the political attitudes of individuals do not follow the normal frequency distribution ….” [80], p. 73. This attitude is still *en vogue* today, see, for example, [81]. We see no need for such a restrictive assumption, and we use four distinct distributions to generate simulated respondent positions on the underlying dimension:

1. A normal distribution—N(50,20);
2. A skewed normal distribution (location parameter: 15, scale parameter: 25, shape parameter: 5), see [82].
3. A bimodal distribution defined by the mixture of two normal distributions—N(25,10) and N(75,10);
4. A uniform distribution on the interval from 0 to 100;

The uniform distribution is defined on the interval between 0 and 100; the same 0 to 100 interval also contains almost all simulated respondent positions for the three other distributions. Fig. 1 depicts graphically a single such sample for each of these distributions.

Obviously, the underlying distributions differ not only qua ‘shape’, but also in terms of variance (which is smallest in the skewed normal case, and largest in the uniform one). When we refer to these distributions by their shape, it should therefore be kept in mind that they also differ in their variances. We will highlight this where this may help to understand differences in findings for the different distributions. All simulated samples have a length of 2000, which reflects the sample size of many contemporary commercial, academic and omnibus surveys. It is important to note that the findings we report below, are not driven by this choice of sample size, and are equally pertinent for smaller sample sizes, as are more common in some fields of research. This is true both for statistics that are relatively robust to variations in sample size (such as AGFI and RMSEA) as well as for those that are significantly affected by sample size (chi square). In the latter case, the alpha we chose is such that no results are driven by an ‘overpowering’ effect of a large sample.

We subsequently define a pool of Likert-type (ordered-categorical) items on the dimension, by specifying for each the boundaries on the continuum that separate the response categories. We defined a pool of 27 items, from which 5, or 8 or 10 items are randomly drawn for every data set to be simulated. As in other simulation studies (cf. [2,35]), items are defined by the location of the four boundaries that separate the five categories of a Likert item. Where these boundaries are located is immaterial in view of our strategy to sample items from the pool, and as long as the entire pool contains sufficient variation between items. Any other pool of items with similar variation in these boundaries would therefore have yielded similar results as those reported in this article. Table 1 describes the pool of items and demonstrates that the required variation in the locations of boundaries between response options is available.

**Table 1:** Specification of boundaries between categories of five-point ordered categorical items*.

Item	Category boundary 1	Category boundary 2	Category boundary 3	Category boundary 4
Item 1	13	21	29	36
Item 2	16	24	33	41
Item 3	18	28	36	46
Item 4	21	31	38	48
Item 5	24	34	42	53
Item 6	27	38	46	56
Item 7	31	41	48	59
Item 8	34	45	55	64
Item 9	36	48	57	66
Item 10	41	51	61	69
Item 11	45	54	64	73
Item 12	48	57	66	76
Item 13	53	62	71	79
Item 14	56	66	75	83
Item 15	61	70	79	86
Item 16	18	29	41	56
Item 17	24	38	51	62
Item 18	31	44	56	71
Item 19	38	51	63	77
Item 20	41	54	68	83
Item 21	25	41	54	66
Item 22	26	46	64	81
Item 23	21	46	66	83
Item 24	25	51	71	81
Item 25	24	44	65	84
Item 26	19	26	53	82
Item 27	21	38	51	59

* The first response category captures all cases with positions between minus infinity and up to (but not including) the first category boundary. Similarly, the fifth response category begins at the value of the fourth category boundary and stretches to plus infinity.

Having defined a population distribution and a set of items, the simulated response to an item is determined in first instance deterministically by the location of the respondent position in terms of the category boundaries of the item (see Table 1). Thus, in an errorless world, a respondent, whose position on the latent dimension is, for example, 33, would give a response of 4 to item 1, as 33 is higher than boundary 3 and lower than boundary 4 for that item. That same respondent would, however, give a response of 1 to item 12, as 33 is below that item’s first boundary.

These error-free responses can be considered to be the ‘true scores’ for each of the items as known in classical test theory. Such scores represent the inherently unknowable values underlying real-world data. These data are unrealistic, because real world data always has an associated error of some kind. Moreover this kind of error-free data can generally not be factor analysed owing to their resulting correlation matrices being computationally singular (or not being invertible). The simulated data can be made more realistic by adding ‘error’ to the scores; a process that also allows us to vary the degree of error, and to assess whether the capacity of factor analysis to correctly model unidimensional data is affected by this. We defined response error at the level of manifest responses. The direction and magnitude of the error is driven for each item and each respondent separately by a random draw from a normal distribution N(0,1). The (absolute) magnitude of this random draw determines how far the simulated response differs from the ‘true’ score, which requires it to be transformed to a discrete form. The sign of the random draw determines whether this is upward or downward. As the items have only 5 ordered response options, the effect of error is truncated where necessary in order to avoid simulated responses outside the range the available response options. We distinguished a ‘large’ and a ‘small’ error condition as follows. The ‘large’ error condition discretised the random draws as follows: no deviation from ‘true’ response for │z│ ≤ 1; a deviation of 1 response category for 1 < │z│ ≤ 2; a deviation of 2 categories for 2 < │z│ ≤ 3; and a deviation of 3 categories for 3 < │z│. This implies that the simulated response differed from the ‘true’ response in slightly less than one out of three instances. For the ‘small’ error condition the respective cutoff values were 1.2, 2.2 and 3, which implies that simulated responses differed from the true scores in slightly less than one out of four instances. This specification of the error has a number of advantages: (1) it guarantees independence of errors across items; (2) it applies the same discrete random error distribution to all items; (3) it does not alter the sampled distribution of latent respondent positions; (4) it mimics the ‘lumpy’ character of response error in ordered-categorical items; (5) it specifies error in ordinal terms, in line with the character of Likert items; (6) it mimics the intrinsically smaller likelihood in ordered-categorical items of deviating multiple categories from the true response rather than one or none, irrespective of the spacing of the category boundaries.

Obviously, this discretisation function can be specified in many different ways. In order to represent the most common notion of random error, however, it should be symmetric around 0. In order to link the discrete and ordinal random error to the common interpretation of normally distributed error in metric space, the discretisation function should specify decreasing probabilities for increasing ordinal perturbations from the true score. We have no reason to believe that differences in this specification matter for the findings reported below, particularly in light of the relatively minor impact of error levels on our findings reported in the section on conditions driving the risk of over-dimensionalisation. We included the technical implementation in the form of the data generation R script file, S2 Appendix. If desired, this script can easily be adapted to generate new data, implementing other assumptions about the response process and its error component.

Table 2 reports the average and range of the test-retest correlation for the items at each of these error levels. These have been estimated as test-retest correlations based on independently generated responses to all 27 items in the pool for a single simulated sample of respondents (n = 2000.) In view of the discussion earlier in this article, we expressed this test-retest stability in terms of both Pearson and polychoric correlations.

**Table 2:** Range and average test-retest correlations (Pearson and polychoric) for the 27 simulated items, by population distribution and level of random error.

Large Random Error								
	Normal		Uniform		Skewed Normal		Bimodal	
	Pearson	Polychoric	Pearson	Polychoric	Pearson	Polychoric	Pearson	Polychoric
Mean	0.80	0.85	0.88	0.92	0.72	0.77	0.88	0.92
Minimum	0.63	0.68	0.80	0.79	0.44	0.49	0.78	0.82
Maximum	0.87	0.91	0.91	0.98	0.84	0.89	0.93	0.96
Spread	0.24	0.23	0.11	0.19	0.40	0.40	0.15	0.14

Small Random Error								
	Normal		Uniform		Skewed Normal		Bimodal	
	Pearson	Polychoric	Pearson	Polychoric	Pearson	Polychoric	Pearson	Polychoric
Mean	0.85	0.90	0.91	0.95	0.77	0.84	0.92	0.95
Minimum	0.73	0.77	0.87	0.90	0.51	0.62	0.85	0.89
Maximum	0.90	0.94	0.93	0.97	0.88	0.93	0.95	0.98
Spread	0.17	0.17	0.06	0.07	0.37	0.31	0.10	0.09

Table 2 demonstrates some interesting phenomena. Not surprisingly, the mean (across all 27 items) of the test-retest correlations is higher with small random error. Also not surprisingly, this mean is higher for uniform and bimodal distributions than for normal and skewed-normal ones, on account of the larger variance of item scores in the bimodal and uniform population distributions which is generated by the larger variance of respondent positions in these distributions. More interestingly, the test-retest correlations differ considerably between items, as evidenced by the spread of the coefficients (for the same reason as above, this spread is smallest for the uniform and bimodal distributions). As the error generating mechanism is exactly the same for all items, the only reason for these differences is the categorical nature of the items. Although not reported here, the inter-item correlations (either Pearson or polychoric) within each of the distributions differ widely between pairs of items, in spite of the fact that all items reflect the same underlying dimension and that they are subject to exactly the same error-generating mechanism. This is yet another illustration of the inappropriateness of Cronbach’s alpha or similar measures of reliability as an indicator of ‘internal consistency’ or of unidimensionality, as convincingly argued by various authors (cf. [83,84,85]), yet often ignored in practice.

**Fig 2:**
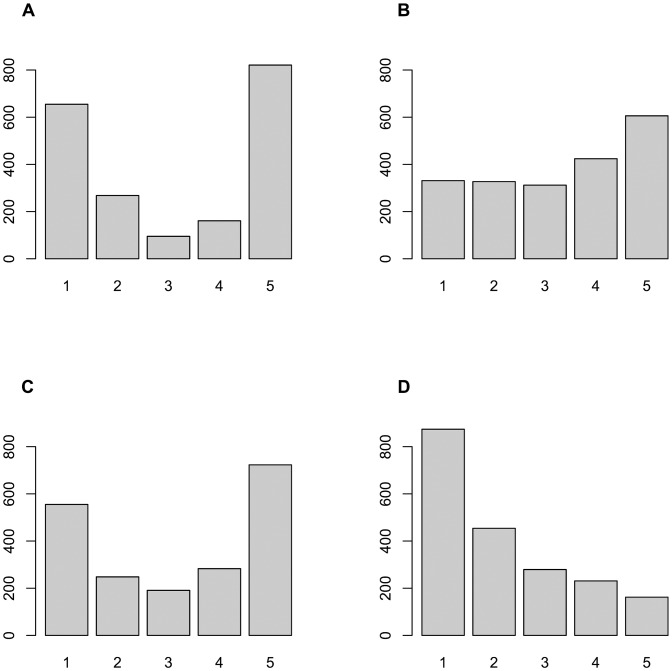
Response distributions for Item 7 for each of the population distributions. Distribution of simulated responses on item 7 (see Table 1) under the large error condition (see Table 2) for four latent population distributions: bimodal (A), normal (B), uniform (C) and skewed normal (D); (n = 2000).

**Fig 3:**
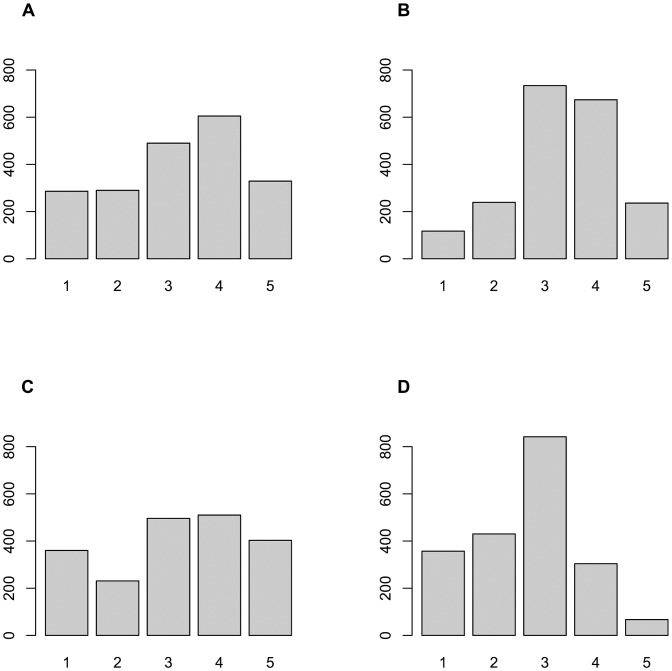
Response distributions for Item 26 for each of the population distributions. Distribution of simulated responses on item 26 (see Table 1) under the large error condition (see Table 2) for four latent population distributions: bimodal (A), normal (B), uniform (C) and skewed normal (D); (n = 2000).

The procedure for generating simulated responses leads, for each of the items, to different response distributions for each of the four population distributions. In Figs. 2 and 3 we report these response distributions, for illustrative purposes, for two items only (items 7 and 26 respectively, see Table 1), in this case calculated under the large error condition. These graphs serve three purposes. First, they demonstrate that our simulations result in ‘realistic’ response distributions that resemble what one could find in many actual surveys. Secondly, these distributions demonstrate, rather dramatically, the well-known but occasionally ignored fact that for ordered-categorical items the observed response distributions are not in any way, shape or form indicative of the form of the underlying population distribution. Indeed, a normally distributed population does not necessarily result in more or less normally distributed observed response distributions, as item 7 clearly shows; nor does a bimodal population distribution necessarily produce bimodal response distributions, as is shown by item 26, and so on. Thirdly, therefore, these illustrative graphs also highlight the impossibility for ordered-categorical items to follow assumptions of normality (let alone multivariate normality). Assumptions of normality are close to ubiquitous; they underlie polychoric correlations; maximum likelihood extraction, and a variety of measures of model fit (either in EFA or CFA). However, normality is for ordered-categorical items not a characteristic of an item (nor is multivariate normality a characteristic of a set of items), because what may appear to be normal in one population may be distinctly not so in another one, as illustrated in Figs. 2 and 3.

### Simulating data-sets, their analyses and harvesting of results

Having defined populations, items, and the response process (including random error) simulated datasets were generated. We varied the number and the identity of the items, by randomly drawing 5, 8 or 10 items from the pool of 27 items described in Table 1. As a consequence, all datasets with the same number of items differ in the identity of those items, thus providing variance in terms of item-related characteristics such as the skew of the items, the spread of their central tendencies, and so forth. A total of 2400 datasets were generated, 100 each for every combination of population distribution (4 distributions), number of items (5, 8 or 10 items) and level of random error (2 levels).

Each simulated dataset was factor analysed in various ways, which reflect actual practice in applied research or methodological recommendations as summarised above. As discussed earlier, our focus is exclusively on the number of factors that researchers would identify on the basis of various criteria. The results of these analyses were harvested to populate a new data-file in which each of the 2400 datasets constitutes a ‘case’ and each of the harvested results a variable. In a small number of cases the algorithms calculating polychoric correlations, or estimating the CFA model did not return results owing to non-convergence of procedures or non-positive definite matrices. In the analyses reported below these missing values are omitted, and thus the n varies slightly between different analyses. We have no reason to believe that this has a systematic impact on our results.

The data set with harvested analysis results (available as S4 Appendix) is the basis for our analyses reported in the following section, where we assess the risk of over-dimensionalisation of different procedures, and where we analyse this risk as a function of the different conditions used in the data simulation.

All factor analyses were conducted using both Pearson correlations and polychoric correlations (the latter were calculated with the R package *psych* [86], calling the R package *polycor* [87]). EFA analyses were conducted with the *fa* component of R package *psych*, using OLS to arrive at a minimum residual solution. This procedure is recommended by Revelle ([86] p.104) as one of the better options, even for badly behaved matrices. In line with our discussion above of such criteria we assessed the dimensionality of the data in exploratory factor analyses (EFA) as follows:

- K1 (eigenvalues ≥1);
- parallel analysis (using the R package *nFactors* [51]), with the number of factors determined by the 95^th^ centile of distribution of eigenvalues of random variables;
- scree-test, assessed non-graphically via the acceleration factor (using the R package *nFactors* [51]);
- improvement in fit (in chi-square) between a 1-factor and a 2-factor model; the 2-factor model would be chosen if it would fit the data significantly better than a 1-factor model. The relevant test consists of the difference of the chi-square fit values of nested models (so-called delta chi-square), which itself is chi-square distributed with degrees of freedom (df) given by the difference of the df of the nested models (delta df);
- RMSEA of the 1-factor model; we applied the commonly used rule of thumb that values in excess of 0.10 indicate poorly fitting models, which should be rejected. This rule holds that RMSEA values below. 05 are seen as acceptable, and values in excess of 0.10 as indicating poor fit (cf. [70]). Instead of using a single cut-off value one could also apply a test of close fit [88], or a test of not-close fit, also known as RMSEA-LB [69] of the 1-factor model. These tests assess whether or not the confidence interval of the RMSEA is in its entirety located below a specified criterion (close fit) or above such a criterion (not-close fit). When applying the latter (not reported here) we find very similar results as presented in Tables 3, 4 and 5.

**Table 3:** Percentage of over-dimensionalised solutions from exploratory factor analysis applying criteria listed in columns; based on Pearson and polychoric correlations of uni-dimensional simulated data; separately for different underlying population distributions and different numbers of items (each cell based on c.200 simulated datasets*).

Latent population distribution	# of items	K1 (eigenvalues>1), Pearson	K1 (eigenvalues>1), Polychoric	Parallel Analysis, Pearson	Parallel Analysis, Polychoric	Acceleration.Factor, Pearson	Acceleration.Factor, Polychoric
Normal	5	2	0	2	0	0	0
	8	23	1	13	0	0	0
	10	55	6	36	5	0	0
Skewed normal	5	6	0	3	0	0	0
	8	32	14	18	6	0	0
	10	63	33	43	16	0	0
Uniform	5	0	0	0	0	0	0
	8	4	0	1	0	0	0
	10	11	0	3	0	0	0
Bi-modal	5	0	0	0	0	0	0
	8	0	0	0	0	0	0
	10	0	0	0	0	0	0

* In a small number of cases the algorithms calculating polychoric correlations did not return results owing to non-positive definite matrices. These missing values are omitted, and thus the n varies slightly between different analyses. We have no reason to believe that this has a systematic impact on the results.

**Table 4:** Percentage of instances where an exploratory 1-factor model would be rejected owing to poor fit according to criteria listed in columns; based on Pearson and polychoric correlations of uni-dimensional simulated data; separately for different underlying population distributions and different numbers of items; (each cell based on c.200 simulated datasets, see also footnote at Table 3).

Latent population distribution	# of items	Chi2 comparison of 1- and 2 factor models, Pearson	Chi2 comparison of 1- and 2-factor models, Polychoric	RMSEA, Pearson	RMSEA, Polychoric
Normal	5	98.5	98	91.5	87
	8	100	100	97.5	96.5
	10	100	100	98.5	99
Skewed normal	5	97	99	90	91
	8	100	100	90	94.5
	10	100	100	96	97.5
Uniform	5	100	100	98	97.5
	8	100	100	100	100
	10	100	100	98.5	98.5
Bi-modal	5	99	99.5	95	97
	8	100	100	98	99
	10	100	100	99.5	100

**Table 5:** Percentage of instances where a confirmatory 1-factor model would be rejected owing to poor fit according to criteria listed in columns; based on Pearson and polychoric correlations of uni-dimensional simulated data; separately for different underlying population distributions and different numbers of items; (each cell based on c.200 simulated datasets*).

Latent population distribution	# of items	CFA Chi2, Pearson	CFA Chi2, Polychoric	CFA AGFI, Pearson	CFA AGFI, Polychoric	CFA RMSEA, Pearson	CFA RMSEA, Polychoric
Normal	5	99.5	97.5	72.5	63	91.5	78
	8	100	97.5	97.5	90.5	97.5	92
	10	100	99	98.5	97	98.5	95
Skewed normal	5	98	97	66	63.5	90,5	81.5
	8	100	94.5	86.5	85.5	90	87.5
	10	100	86	97.5	84	96	81
Uniform	5	100	99.5	86.5	86	98	94.5
	8	100	98	100	98	100	98
	10	100	98	100	94.5	100	94.5
Bi-modal	5	99	97	81	81.5	95	89.5
	8	100	90.5	97	88.5	98	89
	10	99.5	82.5	99	82.5	99	82

* In a small number of cases the algorithms calculating polychoric correlations, or estimating the CFA model did not return results owing to non-positive definite matrices or non-convergence of procedures. In the analyses reported below these cases are omitted, and thus the n varies slightly between different analyses. We have no reason to believe that this has a systematic impact on the results.

All datasets were also subjected to a confirmatory factor analysis (CFA) using a 1-factor model specification. These analyses were conducted with the R package *sem* [89], using default maximum-likelihood (ML) estimation. In comparisons with other estimation methods ML has been shown to provide the most realistic indices of fit [90,91]. We used the following model evaluation criteria:

- chi-square of the 1-factor model; the model would be rejected if the chi-square is significant at a chosen alpha level, which we set (conservatively)at. 001;
- the adjusted goodness of fit coefficient (AGFI) of a 1-factor model. This measure reflects the relative improvement of fit of the specified model over a baseline independence model; generally a value of 0.90 is seen as minimum for an acceptable model [92];
- RMSEA of a 1-factor CFA model, used in the same way as for the EFA analyses.

For CFA models we refrain from the kind of model respecification that researchers would usually engage in if the originally specified model were rejected. Such model adaptations could be made in a large number of different ways (e.g., increasing the number of latent variables; omitting one or several items; specifying correlated error-terms; and so on); however, any such adapted model would be invalid given the true structure of the simulated data.

## Results

We present two kinds of results. First, an assessment of the risk of over-dimensionalisation when factor analysing Likert items. This is a descriptive analysis of how often analysts would be inclined to reject a 1-factor model when using the various criteria discussed earlier. Second, we present a multivariate analysis of conditions that affect this risk.

### Risk assessment

Our estimates of the risk of over-dimensionalisation when factor analysing uni-dimensional Likert items are reported in Tables 3, 4 and 5. In each we distinguish according to the number of items and the underlying population distribution used in the data simulations. In these tables we do not differentiate between levels of random error as these affect the percentages shown only marginally. We assess the impact of error in the multivariate analyses in the next section. In Table 3 we report the percentage of over-dimensionalised solutions when conducting an exploratory factor analysis (EFA), and determining the number of factors on the basis of, respectively, K1, Parallel Analysis, and the Acceleration Factor. In Table 4 we report the results when the number of factors is based on statistical evaluation of exploratory factor analyses. Table 5 reports how often confirmatory factor analysis would lead to rejection of a 1-factor model when using eigenvalue-based criteria.

Table 3 shows that the risk of over-dimensionalisation differs strongly across different conditions and procedures, ranging from a low of 0% to a high of 63%. Of the three criteria concerned, the acceleration factor never suggested an over-dimensionalised solution. Although this is encouraging, it is not sufficient to fully endorse this criterion, in view of reports that it is vulnerable to under-dimensionalisation [53]. Our use of uni-dimensional simulated data precludes us to corroborate these claims. Our results demonstrate clearly why K1 is generally seen as a poor choice. Parallel analysis generally performs better than K1, but still leads in many common conditions to high risks of over-dimensionalisation, particularly when using Pearson correlations, or when analysing larger numbers of items.

A second conclusion that can be drawn from Table 3 is that the use of polychoric correlations lowers the risk of over-dimensionalisation in all conditions modelled. Yet, this is not a failsafe solution to the problem, as the risk remains unacceptably high in quite common circumstances, mainly when using K1 or parallel analysis with 8 or more items.

The third conclusion that can be derived from Table 3 is that the risk of over-dimensionalisation depends strongly on the number of items. Generally, the larger the number of items, the larger the risk.

A fourth observation is that skewed normal distribution is most vulnerable to yield over-dimensionalised solutions, followed by the normal, and subsequently by the uniform and bimodal distributions. This reflects the ordering, from smallest to largest, of the variances in these distributions. Further research is needed to establish whether the differences between the four distributions reported in Table 3 are merely the consequence of the variances of these distributions, of their functional form, or of both.

As argued earlier, the eigenvalue-based criteria reported in Table 3 do not assess how well (or how poorly) a factor model represents the empirical data. It is therefore recommended that exploratory factor analytic models are statistically evaluated, or that they are followed up by a confirmatory factor analysis. As discussed earlier, we chose two criteria to assess the fit of exploratory factor models: the delta-chi-square of the comparison of a 1-factor and a 2-factor model, and the RMSEA of the 1-factor model. Using these criteria a 1-factor exploratory factor model would be rejected if the chi-square difference between a 1-factor and a 2-factor model is large and significant. As chi-square criteria (and their differences) increase with sample size, and our data are based on simulated samples of length 2000, we use in this test an alpha of. 00001, which avoids rejecting the 1-factor model too easily. RMSEA is a function of the error of approximation per degree of freedom; higher values thus reflect less well fitting models. As a rule of thumb models with RMSEA > 0.10 are considered to be poorly fitting.

When applying these evaluation criteria to estimated exploratory factor models, shown in Table 4, we find that in the overwhelming majority of cases a 1-factor model would have to be rejected, irrespective whether Pearson or polychoric correlations are used. In an empirical setting, the values that we found for these criteria would necessitate a reconsideration of the single factor solution, which would often lead to a 2-factor model, or in other words to over-dimensionalisation given the true underlying structure of our data. Indeed, under all conditions, the 2-factor model was not only statistically preferable to the 1-factor model, it generally had acceptable or good statistical fit.

What Table 4 does not demonstrate is how poor the fit of these 1-factor models actually is. For the 5-item datasets, the average chi-square discrepancy of the estimated 1-factor model is 510.38 when using Pearson correlations and 535.54 when using polychoric correlations. These averages are reduced to 10.65 and 16.72 in a 2-factor model, a reduction of discrepancy of more than 95%, resulting in quite acceptable values in themselves. For 8-item and 10-item datasets, the discrepancy is reduced by more than 85% when comparing a 1-factor to a 2-factor model. Likewise, the rejection of exploratory 1-factor models on the basis of RMSEA values is not borderline, but compelling. The average RMSEA of the 1-factor models across all conditions is 0.192 when using Pearson correlations and 0.197 when using polychoric correlations, with little difference across the conditions. Were one to fit a 2-factor EFA model, its average RMSEA would reach values often considered as acceptable:. 067 (for Pearson correlations) and. 082 (for polychoric correlations).

Two additional observations can be made on the basis of Table 4. First, the number of items and the shape (and variance) of the underlying population distribution hardly affect the almost universal extremely poor fit of exploratory 1-factor models on sets of truly uni-dimensional Likert items. Second, in terms of statistical model evaluation it makes very little difference whether the exploratory factor analyses are based on Pearson or on polychoric correlations. Indeed, over all conditions jointly, the results are even marginally worse when using polychoric correlations.

Table 5 reports the results of confirmatory factor analyses (CFA) on our simulated data. The use of CFA is widely recommended as a follow up on exploratory factor analyses, and as an explicit test of a hypothesised (or exploratorily discovered) factorial structure. As discussed earlier, we chose three commonly used criteria for evaluating fit, which were applied to a straightforward 1-factor CFA model: the chi-square and RMSEA, evaluated in the same way as for the EFA results above, and additionally the adjusted goodness of fit measure AGFI (for which we use a cut-off of 0.9, with values under this indicating a poorly fitting model that would be rejected). As the true underlying structure of the items contains only one dimension, such 1-factor models should fit well for CFA to be an appropriate procedure for analysing Likert items.

Table 5 shows very similar results as Table 4: in the overwhelming majority of instances we find that a 1-factor CFA model has to be rejected on account of very poor fit. Neither the character of the underlying population distribution (and thus also its variance), nor the number of items, nor the use of a Pearson or a polychoric correlation matters much in this respect. Again, just as in the discussion of Table 4, the lack of fit is not marginal on any of these criteria, but spectacular. In other words, sensible researchers would, when confronted with these results, reject the 1-factor model and adapt it in ways that would seem reasonable, but that would be incorrect in view of the true uni-dimensionality of the items.

### Conditions driving the risk of over-dimensionalisation

The risk of over-factoring depends on a variety of conditions, including the character of the underlying population distribution, the number of items in the analysis, whether Pearson or polychoric correlations are used, and the particular mode of factor analysis that one uses. Beyond these, other conditions have been mentioned in the literature as increasing the risk of over-factoring, particularly the skew of the items and the spread of the item locations. In addition we aim to assess the consequences of the level of random error in the data. In this section we investigate how these conditions jointly affect the risk of over-dimensionalisation.

Ideally we would have liked to conduct a multivariate binary logistic analysis with the binary outcomes reported in Tables 3, 4 and 5 as dependent variables (i.e. whether or not a specific criterion would have led to the rejection of a 1-factor model for unidimensional Likert items). This approach was not feasible, however, because of the extreme skew, and sometimes even total lack of variance in the dependent variables. We therefore chose a different strategy, and focus instead on statistical evaluation criteria as dependent variables. All of these (chi-square, RMSEA and AGFI values) are metric so that they can be analysed by OLS. The chi-square distributed outcome variables (see Models A, B, E and F in Table 6) have been subjected to a cube-root transformation to avoid the heteroskedasticity that would otherwise be present. All models reported in Table 6 are well-behaved in terms of linearity, homoscedasticity and normality of residuals. The regression coefficients show which conditions push these criteria further towards inacceptable values, or towards acceptable values. As independent variables we use:

- The ‘spread’ of the item locations, measured by the inter-quartile range of the interpolated medians of the response distributions of the items. We expect that the larger this spread, the more the dependent variable is pushed toward inacceptable values, reflecting the phenomenon of spurious ‘difficulty’ factors discussed earlier;
- The differences in item skews, measured by their range. A small range of skews reflects similarity of response distributions of the items, while a large range of skews reflects qualitative differences in these distributions. We expect a large range to push the dependent variable toward inacceptable values, reflecting the impact of skew on over-factoring reported in the literature;
- The degree of random error in the simulated data. This is a dummy variables coded 0 for low error and 1 for high error, as described above (see also Table 2 for the effects of this on reliability);
- Dummies representing the different underlying population distributions (reference category: normal distribution);
- Dummies representing the different sizes of the item sets (reference category: 5 items).

**Table 6:** OLS regressions of model evaluation criteria; cell-entries are regression coefficients (n = 2271–2400, see footnote at Table 5)# *.

	Model A	Model B	Model C	Model D	Model E	Model F	Model G	Model H	Model I	Model J
***Intercept***										
	1.80	0.60	0.03	(0.01)	2.08	1.09	1.16	1.24	0.04	(0.00)
***Error level (dummy*, *reference category*: *low)***										
	-0.92	-0.94	-0.03	-0.03	-1.01	-1.10	0.07	0.07	-0.03	-0.03
***Interquartile range of item interpolated median scores (min*. *0*.*02; max*. *3*.*17; avg*. *1*.*01*, *st*.*dev*. *0*.*43)***										
	3.32	3.01	0.10	0.09	3.11	2.80	-0.29	-0.27	0.10	0.09
***Range of item skews (min*. *0*.*26; max*. *4*.*04; avg*. *1*.*83*, *st*.*dev*. *0*.*82)***										
	1.10	1.72	0.03	0.05	1.11	1.65	-0.07	-0.11	0.03	0.05
***Population distributions (dummies; reference category*: *Normal)***										
Bimodal	1.84	3.02	0.06	0.09	1.97	3.07	-0.14	-0.22	0.06	0.09
Uniform	3.00	3.92	0.09	0.11	3.05	3.82	-0.23	-0.28	0.09	0.11
Skewed Normal	-0.48	-0.36	-0.01§	(-0.00)	-0.52	-0.28^#^	0.01~	(-0.00)	-0.01§	(-0.00)
***Size of item-set (dummies; reference category*: *5 items)***										
8 items	2.90	2.76	-0.03	-0.04	3.25	3.27	-0.03	-0.01~	-0.03	-0.04
10 items	4.35	4.13	-0.05	-0.06	4.89	4.87	-0.05	-0.03	-0.05	-0.06
***Adjusted R*** ^***2***^										
	0.902	0.872	0.816	0.758	0.921	0.898	0.846	0.790	0.816	0.762

# Model A: dependent: Cube root of Δ chi-square between 1- and 2-factor EFA model; Pearson correlations

Model B: dependent: Cube root of Δ chi-square between 1- and 2-factor EFA model; polychoric correlations

Model C: dependent: RMSEA of a 1-factor EFA model; Pearson correlations

Model D: dependent: RMSEA of a 1-factor EFA model; polychoric correlations

Model E: dependent: Cube root of chi-square of a 1-factor CFA model; Pearson correlations

Model F: dependent: Cube root of chi-square of a 1-factor CFA model; polychoric correlations

Model G: dependent: AGFI of a 1-factor CFA model; Pearson correlations

Model H: dependent: AGFI of a 1-factor CFA model; polychoric correlations

Model I: dependent: RMSEA of a 1-factor CFA model; Pearson correlations

Model J: dependent: RMSEA of a 1-factor CFA model; polychoric correlations

* all coefficients p<.00001, except when indicated with § (p<.0001), with # (p<.001), with ~ (p<.01). Bracketed coefficients are not significant (p>.01)

Table 6 shows that the independent variables explain the variation in the various fit measures of a 1-factor model very well: all R-square values are in excess of. 75. This demonstrates that the independent variables in the models include some of the major drivers of (lack of) fit of estimated 1-factor models of uni-dimensional Likert items. We also find that results are exceedingly similar for factor analyses based on Pearson or polychoric correlations (this involves the comparison of Models A and B; C and D, E and F; G and H; and I and J).Table 6 leads to the following conclusions with respect to conditions that affect the fit of factor models of unidimensional Likert items:

- normal or skewed normal population distributions (which contain less variance of respondent positions) lead to somewhat better fitting models than bimodal or uniform distributions (which contain more variance). In actual research, however, it is inherently unknown what this distribution is, and analysts have to make their own assumptions about it. More importantly, the effects of the dummies representing these distributions is only additive (diagnostic analyses did not indicate any need for interactions), which implies that the effect of all other independent variables on (lack of) fit is unaffected by the character of the underlying population distribution.
- higher levels of random noise in the data (i.e., lower reliability) lead to somewhat better fitting models than lower levels of noise. This is to be expected as randomness is easily modelled correctly;
- the larger the similarity of response distributions of the items, the better the fit of a factor model. This shows itself in the spread of item locations (also known their ‘difficulties’ or ‘popularities’) and in the differences of their skews. Larger differences in each of these conditions leads to poorer fit of a 1-factor model;
- the number of items affects fit, but differently for different fit statistics. Larger numbers of items worsen model fit expressed in chi-square distributed criteria. In none of these instances is the worsening of fit compensated by the larger degrees of freedom generated by larger numbers of items. Similarly, in CFA models larger numbers of items reduce the AGFI of 1-factor models of uni-dimensional Likert items. For the RMSEA larger numbers of items have a somewhat beneficial effect, other things being the same.
- The effects of the various independent variables do not systematically differ between factor analyses based on Pearson correlations or polychoric correlations. In other words, the conditions that drive lack of fit (and that therefore increase the risk of over-dimensionalisation) are equally important for both.

The results of Table 6 can be used in real-world (non-simulated) applications to approximate the value one might find for fit criteria of a 1-factor model if the items were really uni-dimensional. The number of items, the spread of their locations and their skews are known from the data; other relevant conditions have to be set by assumption. Thus, for example, having 8 survey items, assuming a normal underlying population distribution, observing a spread of item locations of 2, and a range of item skews of 2.5 and using the coefficients of Models C and D in Table 6 would lead us to expect a RMSEA for a 1-factor EFA model of 0.31 (when using Pearson correlations) or of 0.28 (when using polychoric correlations) for truly uni-dimensional items. These values for spread of item locations and item skews are quite realistic for many real-world surveys. One of the reasons for relatively large values of these spreads is that designers of questionnaires generally try to construct item-batteries in which the items vary strongly in terms of popularity, as that yields more variance of respondent scores, and thus more statistical leverage in subsequent explanatory analyses then when items are all of the same popularity. This expected RMSEA value would be somewhat lower if reliability of the items is low. Such estimates would be particularly useful if one were to find such RMSEA values in actual analysis; they could mitigate the inclination to reject poorly fitting 1-factor models of Likert models without further ado, and encourage further assessment of the latent structure of the items.

## Discussion

This concluding discussion addresses the question of the relevance of our findings for real-world data analysis. We start with a brief discussion of the limitations of our study, and continue by examining the kinds of damage resulting from over-factoring in real-world research. We end with a brief overview of alternatives to factor analytic procedures, and some practical recommendations.

### The boundaries and limitations of this study

#### Limitation 1: we only consider truly uni-dimensional data

Because of our focus on truly one-dimensional data, we can be confident about our conclusion that the sensitivity of factor analysis in the diagnosis of uni-dimensional latent structures is poor. In analysis of non-simulated empirical data, however, we lack certainty about the true latent structure. Therefore we cannot conclude from the present study whether or not the risk of over-factoring also exists for truly two- or higher-dimensional sets of Likert items. Other studies have simulated data from a well-specified true multi-dimensional structure, and have found instances of over-factoring as well as of under-factoring. Owing to often small numbers of simulated data sets, many of these studies serve mainly illustrative purposes while remaining somewhat unclear how typical or atypical their simulated data are, and thus also their findings.

#### Limitation 2: we only consider 5-category Likert items

As stated earlier, the reason for using 5-category items is the popularity of this particular question format. Yet, ordered-categorical items sometimes have fewer options, and sometimes more, and factor analysis is also used on such items. Other studies [31] and smaller scale simulation studies by ourselves, indicate that the problems of over-factoring discussed in the present study exist also when the number of response options is smaller than 5. What remains uncertain, however, is whether or not a larger number of response options help to ameliorate the problems, and, if so, at what number of categories such differences kick in. The scripts used for generating our data can be adapted to investigate this question, and we invite others to do so.

#### Limitation 3: we only consider sets of 5, 8 or 10 items

This limitation is driven by practical consideration, and by the expectation that inter- and extrapolations allow us to formulate reasonable expectations for smaller numbers of items (3 or 4), for sets of 6, 7 or 9 items, and for sets somewhat larger than 10. On the basis of the patterns in Table 6 it is likely that all our conclusions are equally valid for item sets somewhat larger than 10, but without further study it is uncertain whether much larger sets of items (say, in excess of 20) are equally, or less, or more afflicted by risks of over-factoring.

#### Limitation 4: we only consider four ideal-type population distributions

Again, this limitation is motivated by the desire to keep the project manageable, in combination with the inherent impossibility to exhaustively cover all conceivable latent distributions of respondents’ positions. Our study distinguishes itself from most other simulation studies by using a variety of distributions of latent respondent positions, rather than a single one (which then is invariably a normal distribution). Whilst the character of such latent distributions cannot be assessed empirically, they are still of crucial importance. Indeed, even if we were to assume that a latent trait within a population (for example, ‘the adult population of country x’) is normally distributed at the latent level, sampling procedures, exclusion criteria and non-response result in empirical samples that at best represent segments of this population. Crucially, those segments are unlikely to be normally distributed. High non-response, for example, generally leads to empirical samples skewed towards those more interested in the topics at hand. Similarly, self-selection in web-surveys is likely to result in latent distributions of respondent positions that are likely to be skewed normal. In instances where sampling procedures aim to maximise variance, it is likely that the latent distribution of the subpopulation to which the sample can be generalised is bimodal. Our finding that the character of the latent population distribution affects the performance of criteria for model choice thus implies the likelihood of statistical artefacts in all those instances where analysts compare factor structures between different samples.

### The damage resulting from over-factoring

As established in the previous sections, factor analysing uni-dimensional Likert items will in many circumstances lead to a rejection of a 1-factor model. In actual research this would lead to a variety of subsequent decisions by the analyst, all with negative consequences for the validity of knowledge claims and the efficiency of data usage. We briefly discuss four kinds of concerns: conceptualisation, comparability and replicability, reliability, and real-world actions. Finally, we consider a counter argument to these concerns that we frequently encounter, but which is incorrect.

#### Damaging consequences for conceptualisation

The often high risks of over-factoring reported in the previous section imply that in many instances researchers make unwarranted conceptual distinctions. These distinctions may be explicit, which is common when a large item-pools two or more ‘factors’ emerge. Each factor is then interpreted in substantive, conceptual terms. In smaller sets of items the distinction may be implicit: a single factor may stand out, from which only one or few items are excluded. Even when such ‘stray’ items are not explicitly interpreted in conceptual terms, they nevertheless colour the interpretation of dominant factor.

#### Negative consequences for comparability and replicability

The risk of over-factoring is, as demonstrated above, partly dependent on the nature of the underlying population distribution. As a consequence, when samples are compared from populations that are distributed differently on the underlying dimensions, factor analysis will likely yield different results and different substantive interpretations even when the items are uni-dimensional in each of the populations. Analysts will therefore be likely to ‘discover’ different latent structures in different populations, and their creativity will allow them to interpret such differences. This adds a layer of seemingly evidence-based contributions to the literature, which are, in fact, driven by artefactual results. Of course, if different populations have quite similar latent distributions of respondent positions, the likelihood that results can be replicated across these populations is high, however, without any guarantee that what is replicated is actually valid: over-factored results can easily be replicated when observed correlations are very similar, without any of the results necessarily reflecting the actual latent structure.

Such lack of replicability does not have to be wholesale, but may manifest itself in the occurrence of what we referred to above as ‘stray’ items, or in the mapping of individual items on multiple dimensions. Replicability will be endangered when latent population distributions are different, even if the latent structure itself is invariant. This is of particular concern in the context of assessments of invariance of latent structure; cf. [93,94,95].

#### Negative consequences for reliability

In all situations in which truly uni-dimensional sets of items are over-factored, or where some seemingly stray items are deleted from a composite score the resulting measures of latent variables suffer in terms of reliability and, generally, discriminatory power. Although this kind of damage is in our view not the most detrimental of all negative consequences of the inappropriate use of factor analysis on ordered-categorical items, it nevertheless involves a poor use of the scarce good that empirical data are.

#### Negative consequences for real-world actions

The damage to conceptual frameworks (and thus to theories), to comparability and replicability and to reliability (and hence to statistical inference) should not be dismissed as only of concern to academics. Factor analysing Likert items has become commonplace in many fields in which ill-founded results may lead to dangerous consequences when practical actions are based on evidence that is likely to contain artefacts. Important areas of medical practice—particularly in psychiatry, nursing and palliative care—rely on research using questionnaire information, often in the form of Likert items, for diagnoses and to assess interventions; see, for example, [96,97]. Marketing analytics and behavioural economics also make heavy use of questionnaires and Likert items to gauge the perceptions, evaluations, and preferences of consumers and experimental subjects. Here too unjustified conceptual distinctions and poor replicability will cause real damage in terms of costs and revenues. The same holds for much policy relevant research in sociology and politics where, again, invalid results from factor analysing Likert items may lead to policy recommendations that at best are inefficient and at worst counter-productive.

### A counter argument: convergent and discriminant validity

Notwithstanding the results of this article, it may be objected that the consequences of over-dimensionalision will not be especially serious in practice. The argument runs that assessments of convergent and discriminant validity [98] will counteract the tendency to over-dimensionalise. When factor analysis of Likert items yields, for example, two factors while the (unknown) latent structure is truly uni-dimensional, the two factor-scores would be similarly related to other variables. Careful analysts will quickly realise that the two factors do not reflect two different latent phenomena, but that they are different manifestations of the same latent variable. Conversely, if the relationships between two factor scores and other variables are quite different in strength, then the distinction between the factors would be justified. This would be a comforting idea, were it not that one of the most formalised and trusted methods to establish convergent validity is confirmatory factor analysis [99,100], and that our analyses above demonstrated that the risk of rejection of the correct model of latent structure when using CFA is very high (cf. Tables 5 and 6). Less formalised attempts to utilise the framework of convergent and discriminatory validity to ‘weed out’ spurious factors obtained from EFA or CFA are based on comparisons of correlations (usually by visual inspection). However, correlations reflect not only the (manifest or latent) relationship that the analyst is interested in, but also the marginal distributions of the items or composite scores. Different marginal distributions easily lead to large (and statistically significant) differences between items with other variables, even when the items all reflect the same latent variable (i.e., when the latent correlations between the items and the other variable are all equal). In other words, the fatal problems in applying the convergent-discriminatory validity framework on ordered categorical items is exactly the same as in factor analyses of such items.

### If not factor analysis, then what?

Our assessment in this paper is that factor analysing Likert items is ‘risky business’ as it carries a high risk of arriving at incorrect diagnoses of latent structure. Yet, understanding the latent structure of a set of empirical items is extremely important to achieve conceptual clarity ([101], pp. 21–45). Researchers hypothesise that the responses to a plethora of survey items may emanate from a smaller number of underlying (latent) attitudes and orientations. Testing such expectations, and constructing composite indexes to measure these latent variables will, if done correctly, strengthen their research, conceptually, theoretically, analytically and practically. When, however, their empirical information is of an ordinal-categorical nature, factor analysis is in many circumstances likely to lead them astray, as demonstrated earlier in this paper.

The question is thus how to assess the latent structure of responses to Likert items. Relatively little can be gained from various methodological refinements of factor analytic procedures that are suggested in the literature. Using, for example, polychoric correlations instead of Pearson ones, does not really help to address the problem of endemically poor model fit of 1-factor models when they would actually be appropriate. Using Parallel Analysis instead of K1 is somewhat of an improvement, but the PA heuristic by itself is vulnerable to over-factoring for larger item sets, and, again, does not address the problem that adequate fit can generally only be achieved by over-factoring.

Rather than ‘tweaking’ exploratory and confirmatory factor analytic procedures in a variety of ways (some of which requiring rather heroic assumptions) we think the solution would rather be found in using models that have been designed expressly to deal with responses of an ordered-categorical kind. A variety of such models exist. At least three should be mentioned here. One class of such models are so-called IRT (Item Response Theory) models, including the Rasch model [102,103], and the Mokken model [104,105]. Relevant overviews of modern item response theory are presented by [106,107]; other useful texts include [105,108,109,110]. Elsewhere we will present results from ordinal IRT analyses (using the Mokken model) on the same 2400 simulated datasets that were used in this article; these results show that the risk of incorrectly diagnosing any of the items as not belonging to the same latent dimension as the other items is 0.7%. A second variety of models are so-called latent class models [111,112]. A third variety consists of so-called generalised latent variable models, which can validly be applied to continuous as well as ordered-categorical items [113]. At a high level of abstraction all these approaches, as well as factor analysis, can be regarded as special instances of the same model. Yet, such a level of abstraction is of little practical use to applied analysts. As some of the leading scholars in this field note, these models may have many conceptual similarities, yet they are also distinguished by different terminology, model assumptions and testing procedures ([114] p. 280).

Unfortunately familiarity with such alternatives is less widely spread than for factor analysis, but that can be remedied by better training of (post)graduate students, as well as of their trainers. Few of these options are implemented in statistical software packages such as SPSS, STATA and SAS, but all are implemented in well-documented software that is available at limited or no cost (sometimes in the form of add-ons for the STATA and R platforms). The use of these methods when analysing ordered-categorical items will provide more valid answers about latent structure than factor analysis.

### Recommendations

The preceding analysis has shown that assessing the latent structure of ordinal data with factor analysis is fraught with risks. Nonetheless, some procedures are clearly riskier than others; indeed some procedures appear to give rise to essentially acceptable risks. Based on this analysis, we provide a series of recommendations for how to conduct factor analysis upon ordinal data.

- K1 should not be used, given available alternatives.
- The acceleration factor does not appear to over-dimensionalise. However, while this is positive, it does not alleviate the concern that it under-dimensionalises (a concern our study is not equipped to dispel); as such it cannot (yet) be recommend.
- Parallel analysis appears to be the best choice of eigenvalue-based criteria for assessing dimensionality, yet it is still quite prone to over-dimensionalisation when using Pearson correlations, and when analysing larger numbers of items.
- Polychoric correlations are to be preferred to Pearson correlations, but are no panacea: with larger numbers of items (≥10) the risk of over-dimensionalisation remains disturbingly high, even when using parallel analysis.
- Statistical evaluations of factor analytic models (both EFA and CFA) with ordinal data should be treated with extreme caution as they are very prone to suggest over-dimensionalised latent structures.
- Because the risks of over-dimensionalisation increase with divergence of response distributions of items (reflected in differences of item locations and skews), factor analysis of ordinal items should be reserved for sets of items with very similar empirical distributions.
- When using eigenvalue-based factor retention criteria (K1 and PA) the risk of over-dimensionalisation increases strongly with the number of items; these procedures should be treated with extreme caution when analysing larger sets of items.

Finally, in view of the above findings, it is advisable to consider as circumspect extant research that evaluates the dimensionality of ordinal data with factor analytic procedures, particularly when such applications are not in conformity with the recommendations above. In a population that has a single peaked distribution on a latent dimension (an almost ubiquitous assumption), a set of 10 Likert items, evaluated with K1 on Pearson correlations (the most common analysis option reported in the literature) will appear to reflect multiple underlying dimensions in over half of all instances, even when the true dimensionality is one. While this is close to a worst-case scenario, such a situation is by no means rare.

## Supporting Information

S1 Appendix Script File 1 in R. README and Data Initialisation (for more information: see comments in the script file).
(R) Click here for additional data file.

S2 Appendix Script File 2 in R. Script File for Data Simulation (for more information: see comments in script file).
(R) Click here for additional data file.

S3 Appendix Script File 3 in R. Script File for Analysis of Simulated Datasets (for more information: see comments in script file).
(R) Click here for additional data file.

S4 Appendix Data File in SPSS format. Harvested Analysis Results for Simulated datasets (n = 2400)—in SPSS format.
(SAV)
